# Feasibility of Automating Fidelity Monitoring in a Dementia Care Intervention

**DOI:** 10.1093/geroni/igaa057.1593

**Published:** 2020-12-16

**Authors:** Nancy Hodgson, Ani Nencova, Laura Gitlin, Emily Summerhayes

**Affiliations:** 1 University of Pennsylvania, Cherry Hill, New Jersey, United States; 2 University of Pennsylvania, Philadelphia, Pennsylvania, United States; 3 Drexel University, Philadelphia, Pennsylvania, United States

## Abstract

Careful fidelity monitoring is critical to implementing evidence-based interventions in dementia care settings to ensure that the intervention is delivered consistently and as intended. Most approaches to fidelity monitoring rely on human coding of content that has been covered during a session or of stylistic aspects of the intervention, including rapport, empathy, enthusiasm and are unrealistic to implement on a large scale in real world settings. Technological advances in automatic speech recognition and language and speech processing offers potential solutions to overcome these barriers. We compare three commercial automatic speech recognition tools on spoken content drawn from dementia care interactions to determine the accuracy of recognition and the guarantees for privacy offered by each provider. Data were obtained from recorded sessions of the Dementia Behavior Study intervention trial (NCT01892579). We find that despite their impressive performance in general applications, automatic speech recognition systems work less well for older adults and people of color. We outline a plan for automating fidelity in interaction style and content which would be integrated in an online program for training dementia care providers.

